# Phase II study of carboplatin, pemetrexed, and bevacizumab in advanced nonsquamous non–small‐cell lung cancer

**DOI:** 10.1002/cam4.1569

**Published:** 2018-06-14

**Authors:** Nicole F. Laslett, SuJung Park, Gregory A. Masters, David D. Biggs, Charles J. Schneider, Jamal G. Misleh, Kathir Suppiah, Pamela S. Simpson, Stephen Grubbs, Timothy F. Wozniak, Michael Guarino

**Affiliations:** ^1^ Helen F. Graham Cancer Center and Research Institute Christiana Care Health System Newark DE USA

**Keywords:** bevacizumab, carboplatin, molecular therapy, nonsquamous non–small‐cell lung cancer, pemetrexed

## Abstract

Lung cancer remains the leading cause of cancer death throughout the world. Despite new chemotherapeutic, immunomodulating and molecularly targeted agents, patients with locally advanced or metastatic disease still have a poor prognosis. This trial looked to combine antiangiogenic therapy with a first‐line cytotoxic chemotherapy doublet, hoping to extend median progression‐free survival (PFS) while minimizing toxicity in patients with advanced nonsquamous non–small‐cell lung cancer (NSCLC). In this single institution, single‐arm study, 51 patients (age >18 yo) were followed from 2007 to 2012. Patients with stage IV nonsquamous NSCLC and patients with recurrent unresectable disease (nonradiation candidates) were eligible. Treatment consisted of carboplatin AUC 5 IV 30‐60 minutes, pemetrexed 500/mg^2^ IV 10 minutes, bevacizumab 15 mg/kg IV (90 minutes 1st dose, 60 minutes 2nd dose, 30 minutes subsequent doses). Treatment was administered every 21 days and planned for 6 cycles, in the absence of disease progression or unacceptable toxicities. Growth factor support was not permitted prophylactically but allowed for toxicities, as were dose reductions. Maintenance treatment for those with stable disease or better consisted of Bevacizumab 15 mg/kg every 3 weeks for up to 1 year. Between November 2007 and March 2012, 51 patients were followed in the phase II trial of carboplatin, pemetrexed, and bevacizumab. Patients were enrolled over a 24‐month period. After the end of treatment visits, subjects were followed at least every 3 months for survival data. The median follow‐up period was 49 weeks (6 weeks to 178), and the median number of treatment cycles was 6 (range, 1‐6). Among the 50 patients assessable for response, median overall survival was 49 weeks (95% CI, 0‐62.7) with median PFS of 28 weeks (95% CI, 0‐132.4). A complete or partial response was seen in 28 (59.5%) patients. Grade 3‐4 treatment‐related adverse events occurred in 9 (17.6%) of 51 patients; the most common were thrombocytopenia (4 [7.8%]) and neutropenia (3 [5.9%]). Three (5.8%) of 51 patients were discontinued because of treatment‐related adverse events (grade 3 diarrhea, thrombocytopenia, dehydration, fatigue, and grade 4 respiratory distress), and 1 patient (1.9%) was found to be ineligible due to anticoagulation use. A novel 3‐drug combination for advanced nonsquamous NSCLC shows promising efficacy with modest toxicity.

## 1 INTRODUCTION

Lung cancer remains the leading cause of cancer death throughout the world. Non‐small cell lung cancer (NSCLC) accounts for the majority (approximately 85 percent) of lung cancers with the remainder as mostly small cell lung cancer.^1^ Despite new chemotherapeutic, immunomodulating and molecularly targeted agents, patients with locally advanced or metastatic disease still have a poor prognosis. Conventional, first‐line cytotoxic chemotherapy (platinum doublets or nonplatinum doublets) has reached a plateau of a median survival of 7‐8 months. This trial looked to combine an antiangiogenic with first‐line cytotoxic chemotherapy doublet, hoping to extend life expectancy, while minimizing toxicity.

The treatment and prevention of lung cancer are major unmet needs that are improved by understanding of molecular origins and evolution of the disease. Major recent advances in the molecular study of the origins and biology of this disease have facilitated the development of molecular techniques and biomarkers for defining cancer risk, prognosis, and optimal therapy aimed at personalized treatment of lung cancer.

Use of targeted therapy with novel agents has attracted significant attention, particularly given their general tolerability and ability to be combined with chemotherapy without significantly enhancing toxicity. Results of a phase 3 trial^2^ had led the FDA to approve the vascular endothelial growth factor monoclonal antibody bevacizumab in combination with standard chemotherapy for previously untreated, advanced non–small‐cell lung cancer. Bevacizumab when combined with carboplatin/paclitaxel has been shown to improve survival for the treatment of NSCLC and is considered a standard front‐line therapy for most patients with newly diagnosed NSCLC. This study attempted to look for an improvement in progression‐free survival (PFS) with the combination of bevacizumab and carboplatin/pemetrexed in newly diagnosed advanced NSCLC.^2^ Overall survival and safety were also assessed.

## 2 METHODS

### 2.1 Study design and participants

In this single institution, single‐arm study, 51 patients were enrolled over a 24‐month period. Patients with stage IV nonsquamous NSCLC including patients with recurrent unresectable (nonradiation candidates) disease were eligible.

Patients were included in the trial based on the following criteria: advanced stage nonsquamous NSCLC, with measurable or evaluable disease. Prior chemotherapy for early‐stage disease with completion at least 6 months prior to study entry was allowed, as was palliative radiotherapy to painful bony metastases if completed >2 weeks prior to initiation of the study treatment and adequate hematopoietic function. Life expectancy of at least 3 months, ECOG performance status 0‐1, age 18 years or higher are required. Patients must have been willing to use appropriate contraception to avoid pregnancy during the study have had normal organ and marrow function (leukocytes >/= 3000/μL, absolute neutrophil count, >/= 1500/μL, platelets >/= 100 000/μL, total bilirubin within normal institutional limits, AST/ALT </= 2.5 × institutional upper limit of normal, creatinine within normal institutional limits or creatinine clearance >/= 45 mL/min/1.73 m^2^); ability to take folic acid, vitamin B12, and dexamethasone; and ability to interrupt NSAIDS 2 days before (5 days for long‐acting NSAIDS), the day of, and 2 days following administration of pemetrexed.

Key exclusion criteria included prior cytotoxic treatment for advanced nonsquamous NSCLC. Other criteria for exclusion were based on the following: prior definitive chest irradiation (ribs and spine metastasis permitted), known brain metastases unless previously resected and radiated, prior treatment with bevacizumab or pemetrexed, history of allergic reactions or sensitivity attributed to compounds of similar chemical or biologic composition to bevacizumab or carboplatin, concomitant chemotherapy, radiotherapy or investigational agents, evidence of bleeding diathesis or coagulopathy, use of anticoagulant agents (warfarin 1 mg by mouth daily permitted for port maintenance, aspirin and NSAIDS permitted), pregnancy, major surgical procedure, urine dipstick for protein </= 2+ or 24‐hour urine protein <500 mg, history of abdominal fistula, gastrointestinal perforation or intra‐abdominal abscess within 6 months prior to Day 0.

The study was approved by the Christiana Care Institutional Review Board. All patients provided written informed consent to participate before study participation.

### 2.2 Procedures

Patients that met inclusion criteria for the study were administered a 3‐drug regimen consisting of carboplatin, pemetrexed, and bevacizumab. The order of the medication administration was at the discretion of the investigator. Treatment consisted of carboplatin AUC 5 IV 30‐60 minute, pemetrexed 500 mg/m^2^ IV 10 minute, bevacizumab 15 mg/kg IV 90 minute (1st dose), 60 minute (2nd dose), 30 minute (subsequent doses) for 6 cycles followed by Bevacizumab maintenance every 3 weeks for up to 1 year. All patients were prescribed folic acid 1 g PO daily beginning at least 5 days prior to the first dose of pemetrexed and continuing daily until 3 weeks after the last dose of pemetrexed therapy. Vitamin B12 (1000 μg) was administered intramuscularly at least 1 week prior to the first dose of pemetrexed and repeated approximately every 9 weeks until 3 weeks after the last dose of pemetrexed. Dexamethasone 4 mg PO was given twice daily on the day before, the day of, and the day after each dose of pemetrexed for rash prophylaxis. Prophylactic anti‐emetics were at the discretion of the investigator; it was suggested that a 5‐HT3 receptor antagonist be used.

### 2.3 Outcomes

The primary endpoint was PFS. Overall survival was also assessed along with safety. Progression‐free survival was defined as the time from treatment assignment to the date of the first documented tumor progression or death from any cause, whichever occurred first. Overall survival was defined as time between the date of treatment assignment and the date of death from any cause.

Secondary endpoints included assessment of tumor response rate and treatment tolerability as measured by adverse events such as grade 4 toxicities, hospitalizations for toxicities, fever and neutropenia events, and clinically significant bleeding/thrombotic events.

For patients to have a complete response (CR), disappearance of all target lesions on CT scan and absence of appearance of any new lesion were required. Partial response (PR) was assessed by at least a 30% decrease in the sum of the longest diameter (LD) of target lesions without appearance of any new lesions. Progressive disease (PD) was defined as at least a 20% increase in the sum of the LD of target lesions or the appearance of one or more new lesions. Patients were considered to have stable disease if the parameters for PR and PD were not met and no new lesions were identified on imaging. Patients who received one or more cycles were evaluable for response.

### 2.4 Role of the funding source

The funder of the study provided pemetrexed and worked with the investigators to design the study and to collect, analyze, and interpret the data. The corresponding author prepared all drafts of the report with input from all co‐authors and editorial assistance. All authors and professional medical writers had full access to all the data in the study, and all authors had final responsibility for the decision to submit for publication.

## 3 RESULTS

Between November 2007 and March 2012, 51 patients with nonsquamous stage IV disease were enrolled and followed in the phase II trial of carboplatin, pemetrexed, and bevacizumab. Of the 51 patients enrolled, 1 patient was withdrawn due to ineligibility. Three other patients were not evaluated for response due to adverse reactions detailed below.

Patients were enrolled over a 24‐month period for treatment visits. After the end of treatment visits, subjects were seen or contacted by a member of the study team every 3 months for survival data. The median follow‐up was 49 weeks (6 weeks to death) Table 1 shows baseline demographic and clinical characteristics of all treated patients.

**Table 1:** Baseline patient demographics and clinical characteristics

Age	Carboplatin/Pemetrexed/Bevacizumab (n = 50)
<65	26 (52%)
>/=65	24 (47%)
Sex	
Male	26 (52%)
Female	24 (48%)
Race	
White	45 (90%)
African American	5 (10%)
ECOG performance status	
0	6 (12%)
1	44 (88%)

A confirmed investigator‐assessed CR was achieved in 1 (2.1%, 95% CI 0‐6.33) of 47 patients. Partial response was achieved in 27 (57.5%, 95% CI 43‐71.9) of 47 patients evaluated. Grade 3‐4 treatment‐related adverse events occurred in 9 (17.6%) of 47 patients; the most common were thrombocytopenia (4 [7.8%]) and neutropenia (3 [5.9%]). Four (7.8%) of 51 patients were not evaluated because of treatment‐related adverse events (grade 3 diarrhea, thrombocytopenia, dehydration, fatigue, and grade 4 respiratory distress) or were found to be ineligible due to treatment dose reduction or anticoagulation use.

Median PFS was 28 weeks with median overall survival of 49 weeks. Of those patients analyzed, PFS at 6 months was >50% (65.2%). By 12 months, PFS was 18.7%. Median PFS analyzed by gender was equivalent, 28 weeks (range 2‐75 weeks) for female participants, and 28 weeks (range 6.4‐58 weeks) for male participants. However, improved median overall survival was noted in female participants, 56.6 weeks (range 7‐147 weeks) as compared to male participants, 39.3 weeks (range 6‐178 weeks). Median PFS appeared similar across groups separated by race: Caucasian participant median PFS 28 weeks (range 4.7‐75 weeks) and African American participants 27.1 (2‐52).

## 4 DISCUSSION

In this study, a novel 3‐drug combination for advanced NSCLC showed promising efficacy with modest toxicity compared to similar regimens evaluating the addition of bevacizumab to chemotherapy regimens^3^, ^4^, ^5^. Molecularly targeted bevacizumab was used in combination with conventional cytotoxic chemotherapy, pemetrexed, and carboplatin, revealing favorable outcomes in this phase 2, single‐arm clinical trial. Over the half of the patients (58%) achieved a PR. There were 27 (53%) patients that received second‐line treatment and 7 (14%) patients that received third‐line treatment.

**Figure 1:**
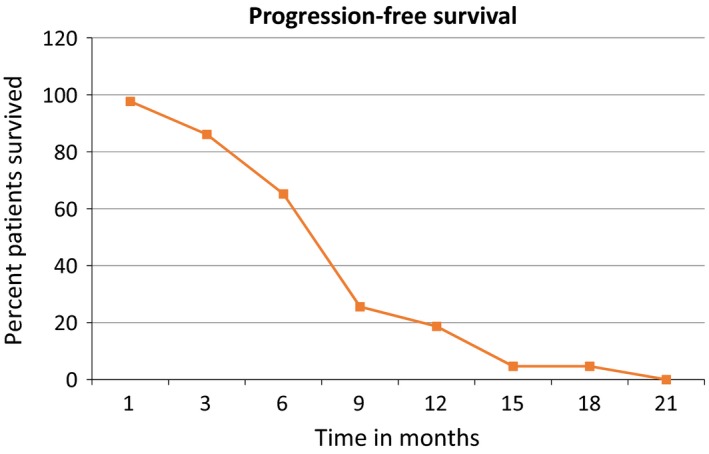
Progression‐free survival across time in months

The primary endpoint showed a median PFS was 7.0 months compared to similar trials^6^ with PFS range 6.0‐7.8 months (Figure 1). In a phase III trial investigating cisplatin/gemcitabine plus bevacizumab^6^ in nonsquamous non–small‐cell lung cancer, median PFS was 6.7 months. In another phase III trial of maintenance bevacizumab with or without pemetrexed after first‐line induction with bevacizumab, cisplatin, and pemetrexed, PFS was improved in the bevacizumab plus pemetrexed arm to 7.4 months. For the patients that achieve disease control with platinum‐based chemotherapy plus bevacizumab, as demonstrated in our trial, bevacizumab plus pemetrexed maintenance may have benefit compared with bevacizumab alone.

**Figure 2:**
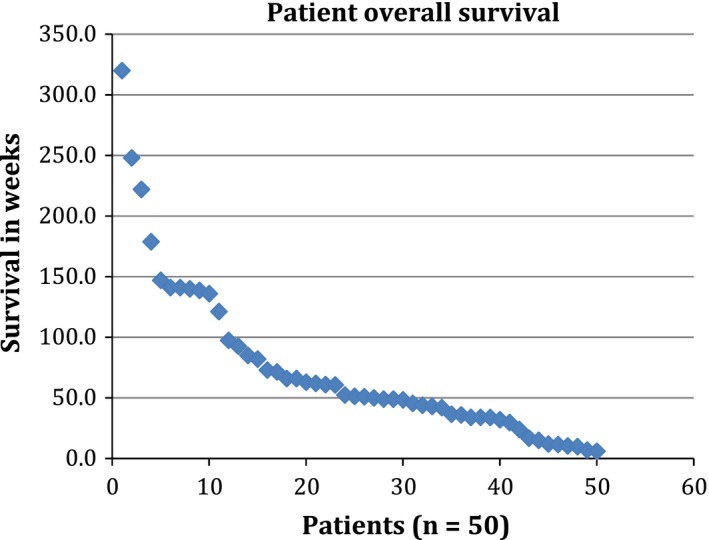
Patient point plot of overall survival in weeks

Overall survival (Figure 2) with this 3‐drug regimen was shown to have an overall median survival of 49 weeks in comparison with other reported trials^7^ of first‐line therapy of combination of pemetrexed plus bevacizumab with median survival of 12.6‐15.2 months. The study's advanced patient age with median age 64 years at enrollment and age 64 years at diagnosis is comparable to national average of 65 years old at diagnosis. The community setting and prolonged follow‐up period are all favorable components of the study.

The study's breakdown between female and male participants also paralleled the national stage. PFS did not vary between genders, but overall survival was 56.6 weeks in females compared to 39.3 weeks in males.

Combination of bevacizumab and carboplatin/pemetrexed was well‐tolerated in this population with limited grade 4 toxicities. Significant toxicities included thrombocytopenia, neutropenia, pulmonary embolism, and anemia. Four patients discontinued treatment because of treatment‐related adverse events (respiratory distress, grade 3 diarrhea, fatigue, and thrombocytopenia), lack of tolerability, or ineligibility (anticoagulation). All deaths of study participants were disease‐related except for 1 (2%): patient suffered from gastrointestinal hemorrhage.

Limitations of the study include the absence of randomization, lack of independent radiology review, and small sample size.

In summary, combination of bevacizumab and pemetrexed/carboplatin was safe with high response rate and encouraging median survival in patients with advanced/ metastatic nonsquamous NSCLC.
